# Environmental Impacts of the U.S. Health Care System and Effects on Public Health

**DOI:** 10.1371/journal.pone.0157014

**Published:** 2016-06-09

**Authors:** Matthew J. Eckelman, Jodi Sherman

**Affiliations:** 1 Department of Civil and Environmental Engineering, Northeastern University, Boston, Massachusetts, United States of America; 2 Department of Anesthesiology, Yale School of Medicine, New Haven, Connecticut, United states of America; University of Alabama at Birmingham, UNITED STATES

## Abstract

The U.S. health care sector is highly interconnected with industrial activities that emit much of the nation’s pollution to air, water, and soils. We estimate emissions directly and indirectly attributable to the health care sector, and potential harmful effects on public health. Negative environmental and public health outcomes were estimated through economic input-output life cycle assessment (EIOLCA) modeling using National Health Expenditures (NHE) for the decade 2003–2013 and compared to national totals. In 2013, the health care sector was also responsible for significant fractions of national air pollution emissions and impacts, including acid rain (12%), greenhouse gas emissions (10%), smog formation (10%) criteria air pollutants (9%), stratospheric ozone depletion (1%), and carcinogenic and non-carcinogenic air toxics (1–2%). The largest contributors to impacts are discussed from both the supply side (EIOLCA economic sectors) and demand side (NHE categories), as are trends over the study period. Health damages from these pollutants are estimated at 470,000 DALYs lost from pollution-related disease, or 405,000 DALYs when adjusted for recent shifts in power generation sector emissions. These indirect health burdens are commensurate with the 44,000–98,000 people who die in hospitals each year in the U.S. as a result of preventable medical errors, but are currently not attributed to our health system. Concerted efforts to improve environmental performance of health care could reduce expenditures directly through waste reduction and energy savings, and indirectly through reducing pollution burden on public health, and ought to be included in efforts to improve health care quality and safety.

## Introduction

The Institute of Medicine 2013 Workshop Summary *Public Health Linkages with Sustainability* suggests that “the health sector should lead by example by greening itself and reducing its ecological footprint….to improve global health and the health of the planet [1].” Quantification of pollution and disease burden stemming from health care is critical to improve the quality and safety of practice, to inform mitigation strategies and leverage health care leadership in sustainable development.

The United States spends the most of any nation by far on its health care system, nearly one-fifth of GDP, or approximately $3 trillion dollars in 2013 [2]. Health care services are also energy-intensive. Hospitals are the second-most energy-intensive commercial buildings in the country, after food service facilities [3]. Hospitals are typically large buildings, open 24 hours a day, seven days a week, and contain several energy-intensive activities, including sophisticated heating, cooling, and ventilation systems, computing, medical and laboratory equipment use, sterilization, refrigeration, laundry, as well as food service [3]. In addition to energy used on site in the form of heating fuels and electricity, the health care system also uses vast quantities of energy-intensive goods and services, such as pharmaceuticals and medical devices, which require significant energy inputs for their manufacturing. As the U.S. is the second-largest emitter of greenhouse gases (GHGs) globally, it follows that the health care sector is an important target for emissions reductions as well. Yet despite its size and status, there has been little work to quantify or probe consumption-based emissions from the U.S. health care sector, how these emissions are trending over time, or how these emissions might affect public health overall.

In 2009, Chung and Meltzer [4] estimated the aggregate carbon footprint of the U.S. health care sector, underscoring the substantial role that health care plays in the physical economy of the country. They report that health care contributes 8% of the nation’s greenhouse gas (GHG) emissions both from health care activities and direct purchases (46%) and from indirect activities associated with the supply chain of health care-related goods and services (54%). Parallel efforts in the United Kingdom report that the National Health Service (NHS) contributes 3–4% of the national GHG emissions total [5]. Efforts are underway to quantify the climate impacts of specific medical devices or supplies and procedures, with the overall objective of finding equally effective but less carbon-intensive ways to deliver care [6–9].

While greenhouse gases (GHGs) are a critical category of emissions and climate change may have severe, negative impacts on human health and livelihoods [10], there are several other categories of emissions from health care with negative environmental and public health consequences that are important to consider. In addition to direct emissions from health care facilities, there are also indirect emissions that occur as a consequence of producing the electricity and materials that those facilities use. In this way, the health care sector is interconnected with and supported by industrial activities that emit much of the pollution to air, water, and soils nationally, including particulate matter, sulfur and nitrogen oxides, persistent organic pollutants, and toxic metals. These very emissions contribute to the national disease burden. Fine particulate matter is the leading cause of air pollution-related disease, with 87% of the world’s population living in areas exceeding the World Health Organization (WHO) Air Quality Guideline of 10 μg/m^3^ PM_2.5_ [11]. The objective of this work is to provide a quantitative estimate of the GHG and non-GHG-related emissions directly and indirectly attributable to the U.S. health care sector in order to assess the scale of potential harmful effects of these emissions on public health.

## Methods

Negative environmental and public health outcomes attributable to the health care sector were estimated for the U.S. using economic input-output life cycle assessment (EIOLCA). Input-output models are compiled by the federal Bureau of Economic Analysis (BEA) and describe monetary flows among all of the 400+ economic sectors that comprise the national economy. EIOLCA extends these economic models by adjoining sector-specific intensity values for emissions and resource use (*e*.*g*., energy, water) per dollar of expenditure. As described in the model documentation [12], for those sectors that report energy use or emissions data, such as utilities or manufacturing, intensity values come from government agencies such as the Environmental Protection Agency (EPA) or Energy Information Administration (EIA). For other EIOLCA sectors for which resource use and emissions are not reported directly, researchers extracted economic data from the BEA Commodity x Industry Use table to determine total expenditures on different fuel commodities (*e*.*g*., coal, oil, natural gas), used average price data to translate these dollar values to physical amounts of fuels, and used emission factors for each fuel to estimate total emissions of different pollutants. Once all intensity values have been adjoined, matrix algebra model algorithms then use economic activity in a given sector to calculate both direct emissions (from that sector) and indirect emissions (from all other linked sectors) that occur throughout the entire economy as a result of that activity [13].

We use health care spending data compiled in the US National Health Expenditure Accounts for the decade 2003–2013 in all categories of health consumption and investment expenditures [2]. Each expenditure category is matched to the corresponding economic sector (S1 Table) in the most recent 2002-vintage purchaser-price EIOLCA model, housed at Carnegie Mellon University [12]. This model requires inputs in nominal 2002 dollars, so expenditures in subsequent years are deflated to this base year using the National Health Expenditures Medical Price Index [2], calculated using the component-based Producer Price Index and Consumer Price Index from the Bureau of Labor Statistics (S2 Table). This approach allows for a dynamic view of health care-related emissions and damages over the study period.

For each expenditure, the EIOLCA model then returns direct and indirect emissions to air, water and soils; these emissions form the life cycle inventory (LCI) of the results. These emissions are then linked to nine categories of environmental and human health outcomes, included in this analysis in order to quantify the contribution of health care-related activities relative to national totals. These impact categories include global warming; stratospheric ozone depletion (allowing higher levels of short-wave ultraviolet light through the atmosphere, increasing the health risks of skin cancer); respiratory disease from inhalation of primary and secondary particulate matter (PM) and from ground-level ozone (smog) stemming from emissions of criteria air pollutants; cancer and non-cancer disease through inhalation and ingestion routes of chemical exposure; environmental effects of acidification (from formation and deposition of acid rain) and eutrophication (algae blooms from excess nutrients) in soils and surface waters; and ecotoxicity that reflects the toxic burden of all emitted chemicals to aquatic organisms. Emissions are linked from the EIOLCA model to these nine categories of environmental and human health impacts using the USEPA’s life cycle chemical fate-exposure-effect model (TRACI) [14]. Each emitted substance that contributes to a particular environmental or health impact is then scaled by its impact-specific potency, represented by a ‘characterization factor’. Each factor is endpoint- and substance-specific and is a complex function of a chemical’s fate and transformation in the environment, chemical activity, uptake and exposure, and potential toxicity. In the public health context, characterization factors measure average health damages per unit of chemical emitted. Characterization factors are relative, measured against a reference substance for which effects are well-known, and so have common equivalent units, such as CO_2_-e for global warming [15].

In life cycle assessment methodology (ISO 14040/44), an optional step is normalization, that is, scaling results by a reference set of values to ease interpretation [16]. Given the national level of our analysis, here we use a normalization set reflecting U.S. totals for each environmental and human health impact category. Health care sector totals are divided pairwise by this normalization set to arrive at the national percentages for each impact category. Normalization sets have been estimated previously for several versions of the TRACI model [17–19]. We make use of the most recent set available, calculated by Ryberg *et al*. [19] for the TRACI 2.1 model for the impact categories of global warming (in CO_2_-e), acidification (in SO_2_-e), criteria air pollutants (in PM_2.5_-e), eutrophication (in N-e), stratospheric ozone depletion (in CFC-11-e), and photochemical smog formation (in O_3_-e) [14]. This normalization set is for year 2008 emissions. We update these to 2013 by scaling with the decrease in GHG emissions from 2008 (7,050 million tons CO_2_-e) to 2013 (6,673 million tons CO_2_-e), for a ratio of approximately 1.06:1. It was not possible to perform normalization for ecotoxicity potential because national estimates have not been reported with units or levels of aggregation consistent with EIOLCA outputs. For human cancer and non-cancer disease, we use estimates from Lautier *et al*. [18] that are expressed using the same reference substances for cancer and non-cancer effects as the version of TRACI implemented within the EIOLCA online tool. These are benzene equivalents for cancer and toluene equivalents for non-cancer health effects. EIOLCA outputs low and high estimates for benzene-e and toluene-e emissions. We use the average of these results. Details of how these benzene and toluene equivalents are calculated in the original TRACI model can be found in Bare [20].

Unit conversion was necessary for several impact categories where the reference substance from the TRACI results did not match the reference substance needed for subsequent analysis. For particulate matter (respiratory inorganics), the output of EIOLCA used PM_10_ as the reference substance, while our normalization set used PM_2.5_. Conversion was performed using the ratio of characterization factors in the TRACI method [20]: PM_2.5_ = 1.67 PM_10_-e. For photochemical oxidation potential (smog formation), the reference substance from EIOLCA is ozone equivalents while the reference substance for the IMPACT2002+ method (used for damage assessment, below) is ethene (C_2_H_4_). Conversion was performed using the ratio of characterization factors in TRACI: C_2_H_4_ = 8.99 O_3_-e. For human health cancer and non-cancer effects, the reference substances from EIOLCA are benzene (C_6_H_6_) and toluene (C_7_H_8_) equivalents while the reference substance for the IMPACT2002+ method is chloroethylene (C_2_H_3_Cl). Conversion was performed using the ratio of characterization factors in IMPACT2002+ for emissions to water: C_6_H_6_ = 0.118 C_2_H_3_Cl-e and C_7_H_8_ = 0.0127 C_2_H_3_Cl-e.

Results for each category are summed across U.S. expenditures to create health care sector totals, which are then compared to U.S. totals [19]. Health care-related results were translated from each emissions equivalents units of CFC-11-e (stratospheric ozone depletion, leading to skin cancer), C_2_H_4_-e (smog formation), PM_2.5_-e (respiratory disease), and C_2_H_3_Cl-e (human health) to the public health metric of disability-adjusted life-years (DALYs) lost using damage assessment factors from the IMPACT2002+ model [21]. Factors used were 1.05×10^−3^ DALYs per kg CFC-11-e, 2.13×10^−6^ DALYs per kg C_2_H_4_-e, 7.00×10^−4^ DALYs per kg PM_2.5_-e, and 2.8×10^−6^ DALYs per kg C_2_H_3_Cl-e emitted. Though health damages from increased ultraviolet radiation, poor ambient air quality, or toxic exposures may take many years to manifest, they are assigned to the year in which the emission took place.

## Results

### GHG Emissions

**Fig 1:**
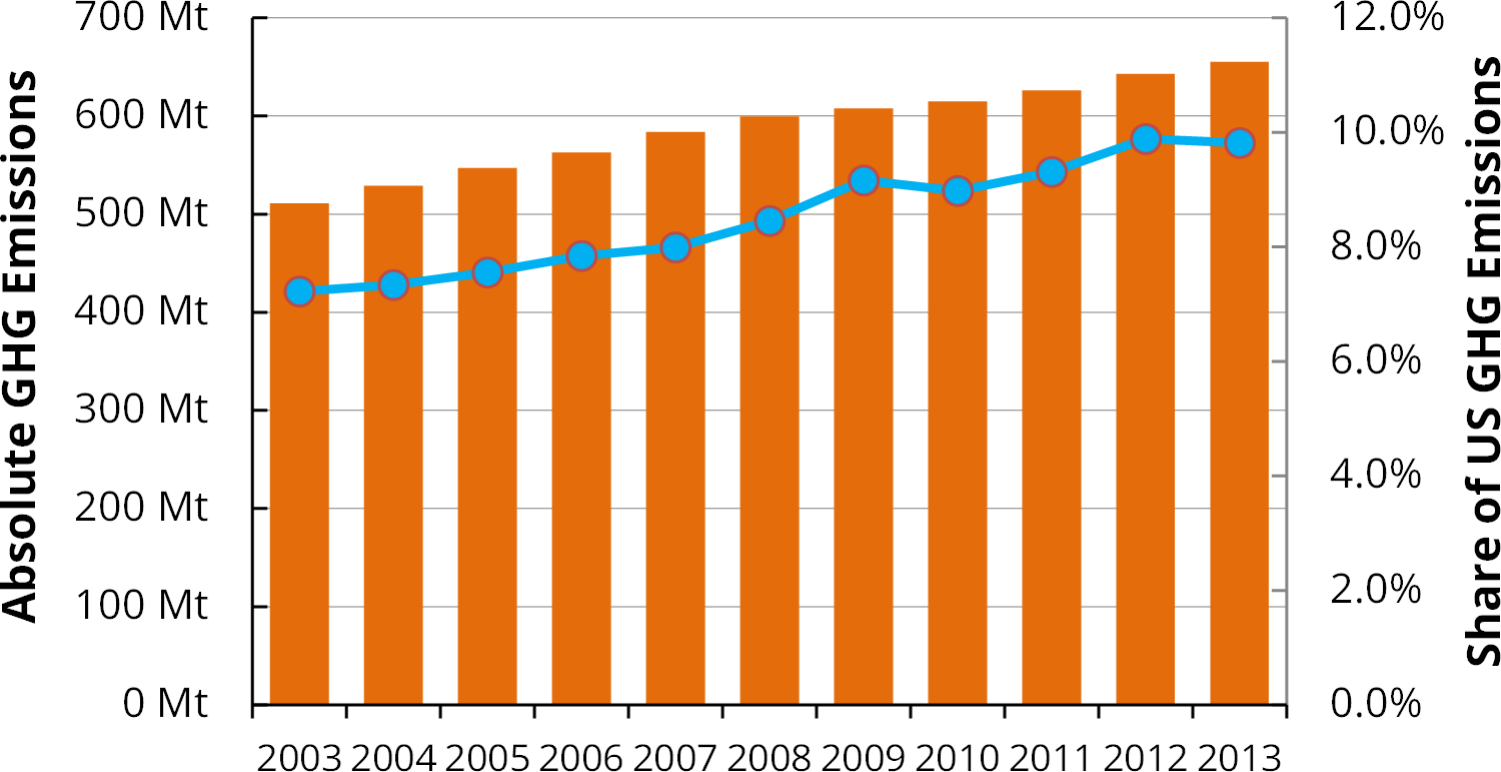
Time series of life cycle GHG emissions from US health care activities. Shown for 2003–2013, in absolute terms (orange bars) and as a share of U.S. national emissions (blue line). Mt = million metric tons.

Fig 1 shows growth in U.S. health care GHG emissions, with an increase of more than 30% over the past decade to a total of 655 million metric tons carbon dioxide equivalents (Mt CO_2-_e) in 2013, or 9.8% of the national total. If the US health care sector were itself a country, it would rank 13^th^ in the world for GHG emissions, ahead of the entire U.K. [22]. Disaggregated results are provided in Table 1. The largest contributors to emissions by expenditure category were Hospital Care (36%), Physician and Clinical Services (12%), and Prescription Drugs (10%), not including releases of waste anesthetic gases. Expressing the results disaggregated by EIOLCA sector reveals that only 2.5% of GHG emissions were directly from the operation of health care facilities (*e*.*g*., from on-site boilers), meaning that the majority of health care’s carbon footprint is associated with its suppliers of energy, goods, and services. Among these supplying sectors, the largest sources of GHG emissions induced by health care activities were: power generation (36%); government services (8%); non-residential commercial and health care construction (this includes “embodied carbon” of health care facilities (4%); and basic organic chemicals manufacturing (3%) (Table A in S1 File).

**Table 1:** Absolute health care greenhouse gas emissions (Mt CO_2_-e) by National Health Expenditure category and U.S. total for 2003–2013

Expenditure category / Year	2003	2004	2005	2006	2007	2008	2009	2010	2011	2012	2013
Hospital Care	184	188	195	200	206	210	218	222	226	233	238
Physician and Clinical Services	57	60	62	65	65	68	69	70	72	74	77
Other Professional Services	7	8	8	8	8	8	9	9	9	10	10
Dental Services	11	12	12	12	12	12	12	12	12	12	11
Other Health, Residential, and Personal Care	20	21	22	22	23	23	24	25	25	25	26
Home Health Care	9	10	11	12	13	13	14	15	15	16	17
Nursing Care Facilities and Continuing Care Retirement Communities	35	36	37	37	38	39	39	39	40	40	41
Prescription Drugs	59	63	65	68	71	71	72	69	68	67	68
Durable Medical Equipment	12	13	14	15	16	16	16	16	17	17	18
Other Non-Durable Medical Products	11	11	12	12	13	13	13	13	14	15	15
Government Administration	13	13	14	14	13	13	13	13	14	14	15
Net Cost of Health Insurance	7	7	7	8	8	8	8	8	8	8	9
Government Public Health Activities	28	28	28	28	29	30	31	31	29	29	29
Research	12	12	13	12	12	12	12	13	12	12	11
Structures and Equipment	45	47	50	51	57	62	59	60	65	70	71
**Health Care TOTAL**	**511**	**529**	**547**	**563**	**584**	**600**	**608**	**615**	**626**	**643**	**655**
**U.S. TOTAL**^a^	7073	7208	7245	7182	7308	7096	6636	6849	6727	6502	6673
**Health Care % of U.S. GHG Emissions**	**7.2%**	**7.3%**	**7.6%**	**7.8%**	**8.0%**	**8.5%**	**9.2%**	**9.0%**	**9.3%**	**9.9%**	**9.8%**

^a^ US national emissions are from the annual US Greenhouse Gas Emissions Inventory conducted by the USEPA.

Several trends are evident in U.S. health care GHG emissions over the past decade. Health care spending has increased monotonically, with a slight decrease in 2011 in real dollars. As emissions are directly proportional to spending in EIOLCA models and are based on a single year (2002 in this case), emissions estimates have also increased over the decade in every spending category. (Adjustments to results based on using dynamic rather than static emission factors are discussed in the Assumptions and Uncertainty section below.) The greatest increases over the decade have been in home health care (+66%) and hospital care (+41%). At the same time, national GHG emissions as inventoried by the EPA have been trending down, largely as a result of efficiency improvements, decreased motor vehicle use, and fuel switching for production of heat and electricity.

### Non-GHG Emissions

Total results for 2013 in all environmental categories are shown in Table 2, with the proportion of national totals in each category and the total disease burden, in DALYs. Through its direct and indirect emissions, U.S. health care was responsible for significant fractions of national air pollution and subsequent public health burdens, including acidification (12%), smog formation (10%) and respiratory disease from particulate matter (9%), with a lower contribution to ozone depletion and carcinogenic and non-carcinogenic air toxics (1–2%). Health damages from these five categories of pollutants are estimated at 470,000 DALYs lost due to health care activities in 2013.

**Table 2:** Environmental and health effects due to health care sector direct and indirect emissions for 2013.

Effect category	Unit / Reference Substance ^a^	Health Care Total	National Total	% of National	DALYs Lost ^e^
GW	kg CO_2_-e	6.6E+11	6.5E+12	9.8% ^c^	-
AP	kg SO_2_-e	3.1E+09	2.6E+10	11.7% ^c^	-
PM	kg PM_10_-e	1.0E+09	6.8E+09	8.9% ^c^	435,000
EP	kg N-e	9.4E+07	6.1E+09	1.5% ^c^	-
ODP	kg CFC-11-e	7.3E+05	4.5E+07	1.6% ^c^	770
POP	kg O_3_-e	4.0E+10	3.9E+11	10.0% ^c^	9,400
ETP	kg 2,4-D-e	6.9E+07	- ^b^	- ^b^	-
HH canc	kg benzene-e	2.5E+08	2.6E+10	1.0% ^d^	84
HH non-canc	kg toluene-e	6.9E+11	3.3E+13	2.2% ^d^	25,300

*Abbreviations*: GW, global warming; AP, acidification potential; PM, particulate matter; EP, eutrophication potential; ODP, ozone depletion potential; POP, photochemical oxidation potential (smog formation); ETP, ecotoxicity potential; HH canc, human health cancer effects; HH non-canc, human health non-cancer effects; suffix–e, equivalents.

^a^ Reference substances from the TRACI model.

^b^ Ecotoxicity national totals not available as reported disaggregated for metal and non-metal emissions.

^c^ Normalization from Ryberg *et al*. [19].

^d^ Normalization from Lautier *et al*. [18].

^e^ Calculated using endpoint characterization factors from IMPACT2002+ model; GHG, AP, EP, and ETP impacts have indirect impacts on human health but robust endpoint characterization factors do not exist.

**Fig 2:**
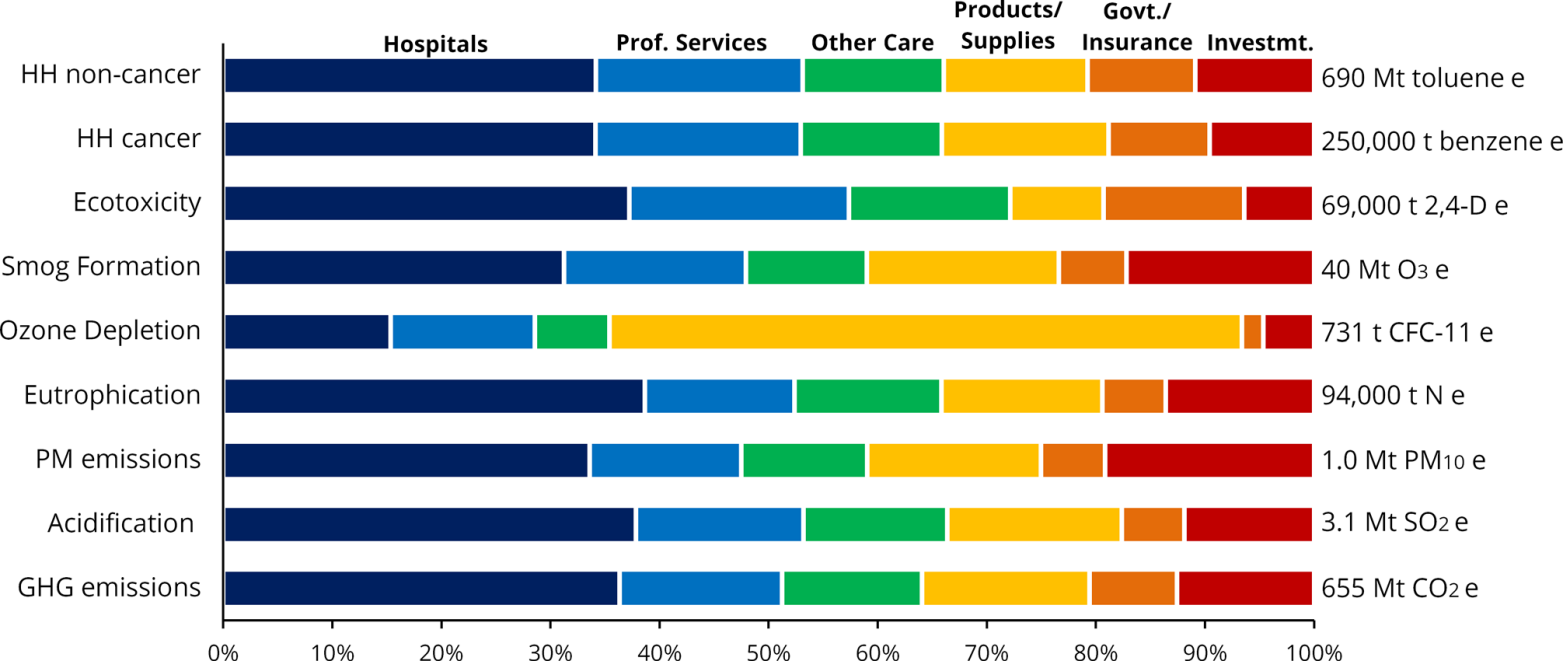
Environmental/health impacts of U.S. health care activities. Depicted by TRACI impact category (left vertical axis) and disaggregated by expenditure categories (colors, horizontal axis). Sector totals listed for each impact category (right vertical axis). Mt = Million metric tons, Prof. = Professional, Govt. = Government, Invstmt. = Investment.

Disaggregated results by health expenditure category are presented in Fig 2, with numerical details provided in S3 Table. In 2013, hospitals were the largest contributor among expenditure categories to environmental and health impacts, between 31–37% of the total. The one exception is for ozone depletion, to which prescription drug expenditures were the largest contributor at 33%, with an additional 22% from medical devices and 15% from hospital care. Health care structures and equipment contribute 17% of PM-equivalent emissions and 15% to smog formation, largely from construction and manufacturing activities. Expressing the results disaggregated by EIOLCA sector (Tables A-I in S1 File) also reveals interesting patterns. ‘Power generation and supply’ is the largest contributing supply sector by far to acidification (44%), respiratory impacts (26%), eutrophication (21%), and smog formation (28%) impact categories, as a result of electricity use in health care facilities and their supply chains. For ozone depletion, however, the largest contributors were ‘Surgical and medical instrument manufacturing’ and ‘Pharmaceutical preparation manufacturing’ (23% each), from the use of halocarbon solvents, refrigerants, propellants, blowing agents, and other ozone depleting substances. For ecotoxicity and human health toxicity (cancer and non-cancer) the most important EIOLCA sector ‘Waste management and remediation’, which contributed more than 85% of the total to ecotoxicity and >50% to human health toxicity impacts.

**Fig 3:**
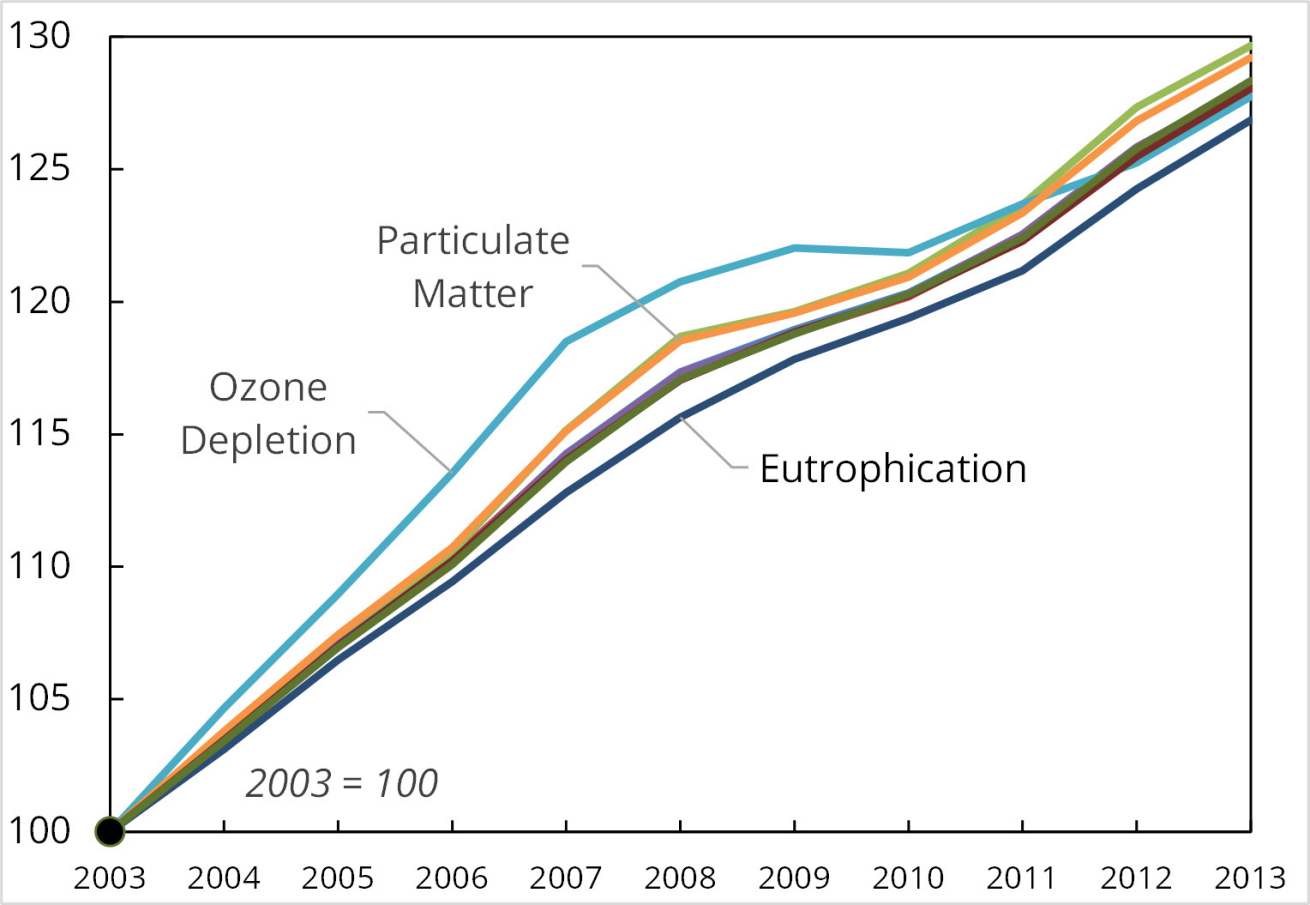
Time series of all life cycle impact impacts from U.S. health care activities. Shown for 2003–2013, in absolute terms.

Fig 3 shows results for all impact categories over the period 2003–2013, with numerical details provided in S4 Table. The overall increase in modeled impacts was remarkably consistent, between 27–30% across all impact categories. Ozone depletion shows the steepest slope (fastest increase) in impacts for the period 2003–2010, reflecting the large increase in ozone depletion-intensive prescription drug expenditures during that period. This increase in expenditures of 78% in real (inflation-adjusted) dollars exceeded that for all other expenditure categories. The economic slowdown during 2007–2010 can be seen in the sharp corrections for ozone depletion and respiratory effects, but after 2010 all impact categories resume consistent growth to 2013.

All of the above results are a product of life cycle emissions or effect intensities (per dollar expenditure, from the EIOLCA model) and dollars expended in each sector (from NHE accounts). The life cycle intensity values alone are presented in S5 Table for each health care-related EIOLCA sector. Per dollar of expenditure, ‘Nonresidential commercial and health care structures’ is the most emissions-intensive sector (on a life cycle basis, including both direct and indirect emissions) for global warming, acidification, respiratory effects, eutrophication, and smog formation, while ‘Surgical and medical instrument manufacturing’ is the most intensive for ozone depletion potential and ‘General state and local government services’ is the most intensive for all toxicity impact categories, again largely due to waste management activities. Across all impact categories, ‘Insurance carriers’ is the least emissions-intensive EIOLCA sector related to health care, as expected for a service sector without major material or energy requirements.

## Discussion

### Patient Health and Public Health

Indirect health damages stemming from health sector pollution are currently unreported and largely unrecognized in health care, and it is useful to compare these results to other estimates of patient health and public health damages. The Institute of Medicine (IOM) first highlighted in 1999, *To Err is Human*: *Building a Safer Health System*, that 44,000–98,000 people die in U.S. hospitals each year as a result of preventable medical errors, at a total cost of $17–29 billion per year [23]. The IOM report highlighted that the level of preventable harm in medicine is unacceptably high, and mostly the result of faulty systems. This report sparked a national exploration into how the health care delivery system could be redesigned to innovate and improve care through formalized patient safety efforts [24] led by the U.S. Department of Health & Human Services Agency for Healthcare Research and Quality (AHRQ). With a conservative average estimate of 10 years of life lost per fatality [25], DALYs from deaths due to preventable medial errors are of the same order of magnitude as the 470,000 DALY lost due to health care-related emissions, calling for similar national attention to the need for prevention of health sector pollution.

It is important to contextualize these findings through comparison with previous work on U.S. health damages from ambient air pollution. Findings from the Institute for Health Metrics and Evaluation (IHME) for the Global Burden of Disease report, showing PM_2.5_- and ozone-related burdens of just over 1.9 million DALYs [26]. Fann *et al*. estimated PM_2.5_- and ozone-related burdens of nearly 2.2 million years of life lost for 2005 (excluding transboundary emissions) [27], while Caiazzo *et al*. estimated that total combustion-related emissions in 2005 caused 210,600 premature deaths from PM_2.5_ and ozone exposure [28]. While the methods used were different from the EIOLCA methods employed in the present work, suggesting caution in making direct comparisons, these past results suggest that the overall contribution of the health care sector to the national burden of disease is significant.

While some level of emissions and subsequent damages to public health are an inevitable consequence of energy use and general economic activity in our current industrial system, Americans spend more than twice as much on health care as other industrialized countries, without commensurate health benefits [29]. Just as some portion of current health care spending is excess (with a midpoint estimate of 34% in 2011 [30]) and so not necessary to maintain a high level of population health, there are opportunities to reduce health care-related emissions and indirect burdens without compromising patient care through waste reduction and efficiency improvements in the actual delivery of care. Indeed, the follow up IOM report, *Crossing the Quality Chasm*: *A New Health System for the 21st Century* [24], described six aims for quality as a systems property. These aims include avoiding injuries to patients from care that is intended to help them, as well as improving efficiency and avoiding waste. Health care pollution itself is a patient safety issue and pollution prevention ought to be included in ongoing efforts to improve health care safety and quality overall.

### Assumptions and Uncertainty

Uncertainty associated with the EIOLCA model structure and construction is discussed in the model documentation and in supporting literature [12, 13]. Recall that the EIOLCA model adjoins three types of data: a matrix of economic flows (compiled by BEA), a vector of emissions for each sector (compiled from various government sources), and a matrix of characterization factors that link emissions to impacts (calculated from the EPA TRACI model). There can be uncertainty associated with values in each of these data sets. Often parameter uncertainty is unquantified because data are reported to the government directly without statistical analysis or sampling. Other sources of uncertainty include: the use of self-reported, incomplete, and/or aggregated data from industry; bias due to reporting thresholds that lead to chronic underestimation of emissions and therefore impacts (most pronounced for toxicity-related impacts); the linear structure of the model (no returns to scale); classification mismatch between BEA sectors and study sectors of interest (NHE categories in this case); and temporal mismatch between the EIOLCA model year and the study year.

Temporal considerations arise largely because of the reliance on a static economic model. Under this assumption, emissions intensities (mass of pollutants emitted per unit of economic activity) remains fixed for each economic sector, even though improvements in efficiencies and pollution control and the mix of technologies employed. (Price changes have been controlled for using the Medical Price Index as noted.) This effect is perhaps most pronounced for the power generation sector, which has experienced a dramatic decrease in the percentage of coal-fired electric generating units powering the grid over the study period, with a concomitant decrease in the carbon emissions intensity of electricity (GHG emissions per unit of electricity generated) of nearly 17% [31]. If we apply this adjustment to the share of health care GHGs from power generation (37%), this leads to a downward revision of results by ~6% to 607 Mt CO_2-_e of health care GHG emissions in 2013, or 9.3% of the US national total. Considering particulate matter, according to the 2014 National Emissions Inventory (NEI), absolute emissions factors for electricity generation have decreased by 55% for primary PM_10_ and 59% for primary PM_2.5_ (including condensibles) [32], equivalent to a 57% and 61% decrease, respectively, in PM emissions factors when accounting for the increase in electricity generation from 2002–2013. Inspection of the EIOLCA results reveals that electric power generation is responsible for just 26% of total health care-related PM emissions (the percentage is 12% for the economy as a whole [32]). Thus, health damages due to PM exposure can be revised downward by approximately 15%, to 370,000 DALYs, bringing the total for all five pollutant damage categories to 405,000 DALYs. Outside of power generation, many other sectors have also experienced changes in emissions factors due to fuel switching, efficiency improvements, and improved pollution controls. For example, the truck transportation sector has experienced increases in fuel economy, improvements in engine and catalytic converter designs, and more widespread availability of ultra-low sulfur diesel fuel. It is not possible at this time to capture changing emissions trends in all of the 400+ sectors of the economy in order to adjust the results, as was done for power generation, but future versions of the EIOLCA model should provide the means to test the sensitivity of the results to changes in emission factors over time.

Other sources of uncertainty include the characterization factors that relate emissions to changes in ambient concentrations and from ambient concentrations to exposure and disease onset. Though this has been an area of intensive modeling and research recently, it is well-known that factors for human health toxicity are among the most uncertain of all impact categories, especially for toxicity stemming from metal emissions [33]. There is also uncertainty introduced by moving between model versions of TRACI and in using the separate IMPACT2002+ model to extend the TRACI results to health endpoints of DALYs lost and cases incurred, particularly for the human health cancer and non-cancer impact categories.

Future work should take advantage of upcoming model updates (to both EIOLCA and life cycle impact assessment methods) to re-run the analysis and investigate the largest contributing expenditure categories and supply sectors. Model updates may also reduce uncertainty for cancer and non-cancer impact categories including additional toxic releases in the EIOLCA inventory as well as updating characterization factors and eliminating the need for inter-model conversions. We believe, however, that the main finding is robust, namely that the health care sector is responsible for a significant proportion of emissions and public health damages in the U.S. Conversely, efforts to improve resource efficiency in health care, through energy efficiency projects or effective waste prevention and management practices, for example, will not only reap economic rewards for health care facilities but will also produce significant indirect public health benefits.

### Improvement Efforts

Environmental stewardship plays a synergistic role in achieving the triple aim set out by the Institute for Healthcare Improvement and adopted by the Centers for Medicare & Medicaid Services, namely better care for individuals, better care for populations, and reducing per-capita costs [34]. Reducing waste can improve both economic and environmental performance without compromising quality of care [30]. Reducing direct and indirect emissions should be considered a key aspect of building a safer health system to improve health care quality and efficiency and reduce unintended adverse effects, both direct and indirect. Decreases in emissions that are attributable to the health care sector will have direct benefits in the U.S. and elsewhere, for example by decreasing the 3.7 million annual fatalities that result from poor ambient air quality worldwide leading to ischemic heart disease, stroke, chronic obstructive pulmonary disease, lung cancer, and acute lower respiratory infections in children [35], or the 34,000 annual cancer cases in the U.S. alone attributable to occupational and environmental exposures [36]. Economic damages from electricity generation emissions alone in the U.S. has been estimated at in excess of $130 billion annually (dominated by health damages) [37], so controlling emissions and reducing demand for electricity could potentially save billions of dollars in health care costs. For GHGs specifically, climate change mitigation efforts have been specifically called for by the WHO and other leading health care bodies [38]. Potential health benefits include reducing the estimated 150,000 annual fatalities that occur worldwide as a result of climate change [39]. Efforts to improve the carbon footprint of health care will also have environmental and health co-benefits, as has been demonstrated for several other sectors including food and agriculture [40], urban transport and land use [41], and household energy use [42].

The World Health Organization notes the health sector, itself, can reap gains from rapid and early adoption of mitigation strategies that improve access to renewable energy, through environmentally friendly operational and building solutions [43]. In the U.S., the Healthier Hospitals Initiative (HHI) (http://healthierhospitals.org/) is a national campaign launched in 2012 to improve environmental health and sustainability in the health care sector. The HHI was organized with Health Care Without Harm, Practice Greenhealth, and The Center for Health Design, and offers tools and resources developed from the Green Guide for Healthcare. The HHI already engages 1,200+ U.S. hospitals actively seeking guidance on the transition to more sustainable operations. The American Hospitals Association also provides a Sustainability Roadmap (http://www.sustainabilityroadmap.org/.) Both offer recommendations to improve the environmental footprint of key areas that reduce both direct on-site and indirect supply chain emissions, including cleaner and more efficient energy use, water conservation, waste reduction, environmentally preferable supply chain management, safer cleaning chemicals, and healthier foods. The Coalition for Sustainable Pharmaceutical and Medical Devices is seeking to develop manufacturing standards for best practices and reporting transparency, guided by life cycle assessment (http://www.sduhealth.org.uk/areas-of-focus/carbon-hotspots/pharmaceuticals/cspm.aspx).

Clinicians play a critical, yet unaddressed role in health care resource use and represent a key opportunity for waste prevention. Seemingly small changes in how medical supplies are utilized or services delivered could have substantial benefits for resource conservation and public health when magnified over this large sector. Efforts such as the Choosing Wisely Campaign (http://www.choosingwisely.org/) offer evidence-based guidance to reduce unnecessary medical tests, treatments and procedures. A critical knowledge gap exists in the medical community regarding the indirect health consequences of wasteful, non-value added practices in all their forms, making resource conservation education and leadership crucial to improving the health system.

## Conclusions

The fundamental tenet of health care practice is ‘*Do no harm*,’ but ironically, the practice of health care itself causes significant pollution, and, consequently, indirect adverse effects on public health. We quantify life cycle emissions of the health care sector, including upstream and downstream activities, and estimate the magnitude of subsequent impacts to human health. We found this amount of disease burden, unreported and largely unrecognized in health care, is similar in magnitude to annual deaths stemming from preventable medical errors first reported in *To Err is Human* [23], which is a topic of national discourse and institutional efforts to improve health care safety. These findings underscore the need to measure, mitigate, and educate on the considerable human health and environmental impacts associated with health care practice itself. Efforts to improve the environmental performance of health care can reduce expenditures directly through waste reduction and energy savings, but also indirectly through reducing the pollution burden on public health, and ought to be included in efforts to improve health care quality and safety.

## Supporting Information

S1 File Health care impacts by EIOLCA economic sector for 2013 (top 25 sectors). (DOCX)

S1 Table Mapping between National Health Expenditure categories and EIOLCA model economic sectors. (DOCX)

S2 Table Medical Price Index by Health Expenditure category, 1980–2013. (DOCX)

S3 Table Proportional contribution to GHG and non-GHG categories by National Health Expenditure category for 2013. (DOCX)

S4 Table Total impacts for GHG and non-GHG categories for 2003–2013. (DOCX)

S5 Table Relative effect intensities of health care-related EIOLCA sectors. (DOCX)
