# Supplementary material for: Environmental Impacts of the U.S. Health Care System and Effects on Public Health
Source: PLoS One. 2016 Jun 9;11(6):e0157014. doi: 10.1371/journal.pone.0157014 (PMC4900601; doi:10.1371/journal.pone.0157014)
Supplement: S1 File — (DOCX) [file pone.0157014.s001.docx]

**S1 File**

**Table A. Health care greenhouse gas emissions by EIOLCA economic sector for 2013 (top 25 sectors).**

**Table B. Health care acidification impacts by EIOLCA economic sector for 2013 (top 25 sectors).**

**Table C. Health care respiratory impacts by EIOLCA economic sector for 2013 (top 25 sectors).**

**Table D. Health care eutrophication impacts by EIOLCA economic sector for 2013 (top 25 sectors).**

**Table E. Health care ozone depletion by EIOLCA economic sector for 2013 (top 25 sectors).**

**Table F. Health care smog formation (ozone) by EIOLCA economic sector for 2013 (top 25 sectors).**

**Table G. Health care ecotoxicity by EIOLCA economic sector for 2013 (top 25 sectors).**

**Table H. Health care human toxicity (carcinogenic) impacts by EIOLCA economic sector for 2013 (top 25 sectors).**

**Table I. Health care human toxicity (non-carcinogenic) impacts by EIOLCA economic sector for 2013 (top 25 sectors).**

**Table A. Health care greenhouse gas emissions by EIOLCA economic sector for 2013 (top 25 sectors)**

| **EIOLCA economic sector** | **GHG Emissions**  **(% of 2013 health care total)** |
| --- | --- |
| Power generation and supply | 36.1% |
| Government services | 8.4% |
| Oil and gas extraction | 4.9% |
| Nonresidential commercial and health care construction | 4.0% |
| Other basic organic chemical manufacturing | 3.1% |
| Petroleum refineries | 3.1% |
| Cattle ranching and farming | 2.8% |
| Waste management and remediation services | 2.8% |
| Truck transportation | 2.5% |
| Iron and steel mills | 2.2% |
| Industrial gas manufacturing | 1.6% |
| Pharmaceutical preparation manufacturing | 1.3% |
| Air transportation | 1.2% |
| Coal mining | 1.2% |
| Pipeline transportation | 1.1% |
| Cement manufacturing | 1.0% |
| Plastics material and resin manufacturing | 0.9% |
| Paper mills | 0.8% |
| Couriers and messengers | 0.8% |
| Petrochemical manufacturing | 0.8% |
| Fertilizer manufacturing | 0.8% |
| Nursing and residential care facilities | 0.8% |
| Grain farming | 0.8% |
| Hospitals | 0.8% |
| Milk Production | 0.7% |
| REST | 15.6% |
| **TOTAL** | **100%** |

**Table B. Health care acidification impacts by EIOLCA economic sector for 2013 (top 25 sectors)**

| **EIOLCA economic sector** | **Acidification**  **(% of 2013 health care total)** |
| --- | --- |
| Power generation and supply | 43.7% |
| Cattle ranching and farming | 3.9% |
| Truck transportation | 3.7% |
| Nonresidential commercial and health care construction | 2.7% |
| Animal production, except cattle and poultry and eggs | 2.5% |
| Other basic organic chemical manufacturing | 2.4% |
| Rail transportation | 2.4% |
| Paper mills | 2.0% |
| Grain farming | 2.0% |
| Milk production | 1.9% |
| Pharmaceutical preparation manufacturing | 1.7% |
| Couriers and messengers | 1.7% |
| Oil and gas extraction | 1.6% |
| Petroleum refineries | 1.6% |
| Poultry and egg production | 1.6% |
| Government services | 1.5% |
| Sugarcane and sugar beet farming | 1.3% |
| Water transportation | 1.3% |
| Other transportation | 1.3% |
| Wholesale trade | 1.0% |
| Cement manufacturing | 0.9% |
| Hospitals | 0.9% |
| Government services | 0.9% |
| Paperboard mills | 0.7% |
| Coal mining | 0.6% |
| REST | 14.1% |
| **TOTAL** | **100%** |

**Table C. Health care respiratory impacts by EIOLCA economic sector for 2013 (top 25 sectors)**

| **EIOLCA economic sector** | **Respiratory Impacts**  **(% of 2013 health care total)** |
| --- | --- |
| Power generation and supply | 26.0% |
| Nonresidential commercial and health care construction | 9.3% |
| Sugarcane and sugar beet farming | 6.6% |
| Grain farming | 6.0% |
| Nonresidential maintenance and repair | 5.0% |
| Truck transportation | 4.8% |
| Oilseed farming | 2.4% |
| Couriers and messengers | 2.2% |
| Cattle ranching and farming | 2.2% |
| Pharmaceutical preparation manufacturing | 2.0% |
| Government services | 2.0% |
| Other nonmetallic mineral mining | 1.6% |
| Paper mills | 1.6% |
| Other basic organic chemical manufacturing | 1.5% |
| Stone mining and quarrying | 1.3% |
| Wholesale trade | 1.2% |
| Fruit farming | 1.0% |
| Petroleum refineries | 1.0% |
| Sand, gravel, clay, and refractory mining | 0.9% |
| Vegetable and melon farming | 0.8% |
| Paperboard mills | 0.7% |
| Rail transportation | 0.7% |
| Water transportation | 0.7% |
| Cement manufacturing | 0.7% |
| Greenhouse and nursery production | 0.6% |
| REST | 17.3% |
| **TOTAL** | **100%** |

**Table D. Health care eutrophication impacts by EIOLCA economic sector for 2013 (top 25 sectors)**

| **EIOLCA economic sector** | **Eutrophication**  **(% of 2013 health care total)** |
| --- | --- |
| Power generation and supply | 21.1% |
| Cattle ranching and farming | 8.0% |
| Truck transportation | 6.7% |
| Animal production, except cattle and poultry and eggs | 5.1% |
| Nonresidential commercial and health care construction | 4.7% |
| Rail transportation | 4.0% |
| Grain farming | 4.0% |
| Milk production | 4.0% |
| Poultry and egg production | 3.2% |
| Couriers and messengers | 3.1% |
| Oil and gas extraction | 2.8% |
| Government services | 2.8% |
| Sugarcane and sugar beet farming | 2.6% |
| Other basic organic chemical manufacturing | 2.3% |
| Other transportation | 2.2% |
| Wholesale trade | 1.8% |
| Water transportation | 1.5% |
| Paper mills | 1.3% |
| Petroleum refineries | 1.0% |
| Pharmaceutical preparation manufacturing | 1.0% |
| Cement manufacturing | 0.9% |
| Coal mining | 0.8% |
| Forest nurseries, forest products, and timber | 0.8% |
| Government services | 0.7% |
| Oilseed farming | 0.7% |
| REST | 12.9% |
| **TOTAL** | **100%** |

**Table E. Health care ozone depletion by EIOLCA economic sector for 2013 (top 25 sectors)**

| **EIOLCA economic sector** | **Ozone Depletion**  **(% of 2013 health care total)** |
| --- | --- |
| Surgical and medical instrument manufacturing | 23.9% |
| Pharmaceutical preparation manufacturing | 23.1% |
| Other basic organic chemical manufacturing | 20.4% |
| All other basic inorganic chemical manufacturing | 7.3% |
| Urethane and Other Foam Product | 4.6% |
| Alkalies and chlorine manufacturing | 4.1% |
| Plastics material and resin manufacturing | 3.5% |
| Aluminum production | 2.3% |
| Artificial and synthetic fibers and filaments | 2.2% |
| Polystyrene Foam Product Manufacturing | 1.0% |
| Synthetic rubber manufacturing | 1.0% |
| Other fabricated metal manufacturing | 0.7% |
| Paper mills | 0.6% |
| Plate work and fabricated structural product | 0.6% |
| Medicinal and botanical manufacturing | 0.5% |
| Air conditioning, refrigeration, and warm air | 0.5% |
| Plastics Pipe and Pipe Fitting Manufacturing | 0.4% |
| Petroleum refineries | 0.3% |
| Petrochemical manufacturing | 0.3% |
| Paperboard mills | 0.2% |
| Other rubber product manufacturing | 0.2% |
| Wholesale trade | 0.2% |
| Dental equipment and supplies manufacturing | 0.2% |
| Electromedical apparatus manufacturing | 0.2% |
| Ornamental and architectural metal products | 0.2% |
| REST | 1.6% |
| **TOTAL** | **100%** |

**Table F. Health care smog formation (ozone) by EIOLCA economic sector for 2013 (top 25 sectors)**

| **EIOLCA economic sector** | **Smog Formation**  **(% of 2013 health care total)** |
| --- | --- |
| Power generation and supply | 27.5% |
| Truck transportation | 9.0% |
| Nonresidential commercial and health care construction | 6.5% |
| Rail transportation | 5.4% |
| Oil and gas extraction | 4.4% |
| Couriers and messengers | 4.1% |
| Government services | 3.7% |
| Other basic organic chemical manufacturing | 3.1% |
| Other transportation | 2.9% |
| Pharmaceutical preparation manufacturing | 2.8% |
| Wholesale trade | 2.5% |
| Water transportation | 2.0% |
| Paper mills | 1.7% |
| Petroleum refineries | 1.3% |
| Cement manufacturing | 1.2% |
| Coal mining | 1.1% |
| Forest nurseries, forest products, and timber | 1.1% |
| Government services | 0.9% |
| Hospitals | 0.8% |
| Postal service | 0.8% |
| Transit and ground passenger transportation | 0.7% |
| Iron and steel mills | 0.7% |
| Paperboard mills | 0.7% |
| Medicinal and botanical manufacturing | 0.6% |
| Waste management and remediation services | 0.6% |
| REST | 14.0% |
| **TOTAL** | **100%** |

**Table G. Health care ecotoxicity by EIOLCA economic sector for 2013 (top 25 sectors)**

| **EIOLCA economic sector** | **Ecotoxicity**  **(% of 2013 health care total)** |
| --- | --- |
| Waste management and remediation services | 85.3% |
| Power generation and supply | 4.0% |
| Copper, nickel, lead, and zinc mining | 1.5% |
| Lime and gypsum product manufacturing | 1.2% |
| All other chemical product and preparation | 0.8% |
| Copper rolling, drawing, extruding and alloying | 0.7% |
| Nonferrous metal (except copper and aluminum) | 0.6% |
| Nonferrous foundries | 0.6% |
| Primary smelting and refining of copper | 0.5% |
| Coal mining | 0.4% |
| Iron and steel mills | 0.3% |
| Ferrous metal foundries | 0.3% |
| Government services | 0.2% |
| Gasket, packing, and sealing device | 0.2% |
| Sugar cane mills and refining | 0.2% |
| Electric power and specialty transformer | 0.1% |
| Other basic organic chemical manufacturing | 0.5% |
| Iron, steel pipe and tube manufacturing | 0.1% |
| Gold, silver, and other metal ore mining | 0.1% |
| Motor vehicle parts manufacturing | 0.1% |
| Plate work and fabricated structural product | 0.1% |
| Petroleum refineries | 0.1% |
| Paper mills | 0.1% |
| Cement manufacturing | 0.1% |
| Plumbing Fixture Fitting and Trim Manufacturing | 0.1% |
| REST | 1.9% |
| **TOTAL** | **100%** |

**Table H. Health care human toxicity (carcinogenic) impacts by EIOLCA economic sector for 2013 (top 25 sectors)**

| **EIOLCA economic sector** | **HH toxicity cancer impacts**  **(% of 2013 health care total)** |
| --- | --- |
| Waste management and remediation services | 50.9% |
| Alkalies and chlorine manufacturing | 19.3% |
| Power generation and supply | 4.7% |
| Copper rolling, drawing, extruding and alloying | 3.3% |
| Nonferrous metal (except copper and aluminum) | 3.0% |
| Copper, nickel, lead, and zinc mining | 2.2% |
| Other basic organic chemical manufacturing | 2.0% |
| Cement manufacturing | 1.8% |
| Iron and steel mills | 1.7% |
| Ferrous metal foundries | 1.5% |
| Coal mining | 1.4% |
| Synthetic rubber manufacturing | 0.8% |
| Petroleum refineries | 0.7% |
| Paper mills | 0.6% |
| Reconstituted wood product manufacturing | 0.5% |
| All other basic inorganic chemical manufacturing | 0.4% |
| Iron, steel pipe and tube manufacturing | 0.3% |
| Paperboard mills | 0.3% |
| Plastics material and resin manufacturing | 0.3% |
| Fertilizer manufacturing | 0.3% |
| All other chemical product and preparation | 0.2% |
| Other pressed and blown glass and glassware | 0.2% |
| Lime and gypsum product manufacturing | 0.2% |
| Surgical and medical instrument manufacturing | 0.2% |
| Pharmaceutical preparation manufacturing | 0.2% |
| REST | 3.0% |
| **TOTAL** | **100%** |

**Table I. Health care human toxicity (non-carcinogenic) impacts by EIOLCA economic sector for 2013 (top 25 sectors)**

| **EIOLCA economic sector** | **HH toxicity non-cancer impacts**  **(% of 2013 health care total)** |
| --- | --- |
| Waste management and remediation services | 57.1% |
| Alkalies and chlorine manufacturing | 14.9% |
| Power generation and supply | 4.3% |
| Copper rolling, drawing, extruding and alloying | 3.5% |
| Iron and steel mills | 3.1% |
| Nonferrous metal (except copper and aluminum) | 3.0% |
| Cement manufacturing | 1.7% |
| Iron, steel pipe and tube manufacturing | 1.5% |
| Ferrous metal foundries | 1.0% |
| Coal mining | 0.9% |
| Other pressed and blown glass and glassware | 0.7% |
| Artificial and synthetic fibers and filaments | 0.6% |
| Plate work and fabricated structural product | 0.5% |
| Primary smelting and refining of nonferrous | 0.5% |
| Plastics Pipe and Pipe Fitting Manufacturing | 0.5% |
| Services to buildings and dwellings | 0.4% |
| Nonferrous foundries | 0.4% |
| Other basic organic chemical manufacturing | 0.4% |
| Copper, nickel, lead, and zinc mining | 0.3% |
| Paper mills | 0.3% |
| Petroleum refineries | 0.3% |
| Fertilizer manufacturing | 0.2% |
| Wiring device manufacturing | 0.2% |
| Lime and gypsum product manufacturing | 0.2% |
| All other chemical product and preparation | 0.2% |
| REST | 3.1% |
| **TOTAL** | **100%** |
