# Supplementary material for: Environmental Impacts of the U.S. Health Care System and Effects on Public Health
Source: PLoS One. 2016 Jun 9;11(6):e0157014. doi: 10.1371/journal.pone.0157014 (PMC4900601; doi:10.1371/journal.pone.0157014)
Supplement: S1 Table — (DOCX) [file pone.0157014.s002.docx]

**S1 Table. Mapping between National Health Expenditure categories and EIOLCA economic sectors**

| **National Health Expenditure Categories** | **Corresponding EIOLCA Economic Sectors** |
| --- | --- |
| Hospital Care | Hospitals |
| Physician and Clinical Services | Offices of physicians, dentists, and other health practitioners |
| Other Professional Services | Offices of physicians, dentists, and other health practitioners |
| Dental Services | Offices of physicians, dentists, and other health practitioners |
| Other Health, Residential, and Personal Care | Healthcare and social assistance |
| Home Health Care | Home health care services |
| Nursing Care Facilities and Continuing Care Retirement Communities | Nursing and residential care facilities |
| Prescription Drugs | Pharmaceutical preparation manufacturing |
| Durable Medical Equipment | Surgical and medical instrument manufacturing |
| Other Non-Durable Medical Products | Surgical appliance and supplies manufacturing |
| Government Administration | General state and local government services |
| Net Cost of Health Insurance | Insurance carriers |
| Government Public Health Activities | General state and local government services |
| Research | Scientific research and development services |
| Structures and Equipment | Nonresidential commercial and health care structures |
