# Supplementary material for: Environmental Impacts of the U.S. Health Care System and Effects on Public Health
Source: PLoS One. 2016 Jun 9;11(6):e0157014. doi: 10.1371/journal.pone.0157014 (PMC4900601; doi:10.1371/journal.pone.0157014)
Supplement: S2 Table — (DOCX) [file pone.0157014.s003.docx]

**S2 Table. NHE Medical Price Index by Health Expenditure category, 1980-2013**

| **Health Expenditure category** | **1980** | **1990** | **2000** | **2002*** | **2003** | **2004** | **2005** | **2006** | **2007** | **2008** | **2009** | **2010** | **2011** | **2012** | **2013** |
| --- | --- | --- | --- | --- | --- | --- | --- | --- | --- | --- | --- | --- | --- | --- | --- |
| Hospital Care | - | - | - | 81.4 | 84.2 | 87.0 | 90.0 | 92.8 | 95.8 | 97.7 | 100.0 | 102.7 | 105.2 | 106.9 | 108.3 |
| Physician and Clinical Services | 28.5 | 56.4 | 75.7 | 80.5 | 83.5 | 86.5 | 89.1 | 91.8 | 94.8 | 97.3 | 100.0 | 102.7 | 104.8 | 106.8 | 108.4 |
| Other Professional Services | 25.8 | 53.3 | 71.0 | 76.3 | 80.2 | 84.1 | 87.3 | 91.1 | 94.2 | 97.1 | 100.0 | 103.0 | 105.2 | 107.8 | 110.2 |
| Dental Services | 38.6 | 70.8 | 84.7 | 86.8 | 88.6 | 90.4 | 92.2 | 93.1 | 96.8 | 97.7 | 100.0 | 102.3 | 103.7 | 104.9 | 105.0 |
| Other Health, Residential, and Personal Care | 29.5 | 57.3 | 77.2 | 82.1 | 84.4 | 86.7 | 89.1 | 91.6 | 94.1 | 98.0 | 100.0 | 102.2 | 103.7 | 104.7 | 106.5 |
| Home Health Care | 20.3 | 40.1 | 66.6 | 71.7 | 75.4 | 79.1 | 83.5 | 87.8 | 92.3 | 97.1 | 100.0 | 102.7 | 105.1 | 107.6 | 111.2 |
| Nursing Care Facilities and Continuing Care Retirement Communities | 29.1 | 52.2 | 75.9 | 80.6 | 83.5 | 86.4 | 88.8 | 90.8 | 93.6 | 97.5 | 100.0 | 103.4 | 106.7 | 109.0 | 111.4 |
| Prescription Drugs | 31.3 | 63.9 | 86.8 | 89.2 | 91.4 | 93.6 | 94.6 | 95.2 | 96.9 | 98.5 | 100.0 | 101.2 | 101.2 | 102.0 | 101.9 |
| Durable Medical Equipment | 27.3 | 46.7 | 70.1 | 76.5 | 79.9 | 83.3 | 86.4 | 88.9 | 93.1 | 96.8 | 100.0 | 102.0 | 104.5 | 105.9 | 106.8 |
| Other Non-Durable Medical Products | 18.3 | 45.9 | 72.1 | 81.4 | 84.1 | 86.8 | 89.9 | 93.1 | 94.4 | 96.7 | 100.0 | 104.3 | 108.7 | 110.7 | 113.3 |
| Government Administration | 38.8 | 75.6 | 91.7 | 94.0 | 93.9 | 93.8 | 93.1 | 95.1 | 96.6 | 97.8 | 100.0 | 100.0 | 98.6 | 99.3 | 99.4 |
| Net Cost of Health Insurance | 40.5 | 70.0 | 89.8 | 90.3 | 91.7 | 93.1 | 94.5 | 96.6 | 98.1 | 99.0 | 100.0 | 99.9 | 100.5 | 101.6 | 102.1 |
| Government Public Health Activities | - | - | - | 80.5 | 83.7 | 86.9 | 90.0 | 92.7 | 95.7 | 99.0 | 100.0 | 101.8 | 104.4 | 106.2 | 108.1 |
| Research | - | - | - | 86.1 | 88.3 | 90.5 | 97.5 | 102.3 | 106.0 | 98.6 | 100.0 | 107.5 | 116.5 | 116.5 | 115.5 |
| Structures and Equipment | - | - | - | 77.5 | 80.6 | 83.7 | 87.7 | 91.3 | 94.8 | 99.7 | 100.0 | 102.4 | 105.7 | 107.9 | 109.9 |

* 2002 data not provided, values calculated by difference based on 2003-2004 changes.
