# Supplementary material for: Environmental Impacts of the U.S. Health Care System and Effects on Public Health
Source: PLoS One. 2016 Jun 9;11(6):e0157014. doi: 10.1371/journal.pone.0157014 (PMC4900601; doi:10.1371/journal.pone.0157014)
Supplement: S3 Table — (DOCX) [file pone.0157014.s004.docx]

**S3 Table. Proportional contribution to GHG and non-GHG categories by National Health Expenditure category for 2013**

| **Health Expenditure category** | **Impact category** | | | | | | | | |
| --- | --- | --- | --- | --- | --- | --- | --- | --- | --- |
|  | GW | AP | PM | EP | ODP | POP | ETP | HH canc | HH non-canc |
| Hospital Care | 40% | 41% | 37% | 41% | 22% | 33% | 40% | 38% | 38% |
| Physician and Clinical Services | 11% | 11% | 10% | 10% | 13% | 12% | 14% | 14% | 14% |
| Other Professional Services | 1% | 1% | 1% | 1% | 2% | 2% | 2% | 2% | 2% |
| Dental Services | 2% | 2% | 2% | 2% | 3% | 2% | 3% | 3% | 3% |
| Other Health, Residential, and Personal Care | 4% | 4% | 4% | 3% | 4% | 4% | 5% | 5% | 5% |
| Home Health Care | 2% | 2% | 2% | 2% | 2% | 2% | 3% | 3% | 3% |
| Nursing Care Facilities and Continuing Care Retirement Communities | 7% | 8% | 7% | 8% | 3% | 5% | 6% | 5% | 5% |
| Prescription Drugs | 10% | 11% | 11% | 11% | 26% | 14% | 6% | 8% | 7% |
| Durable Medical Equipment | 2% | 2% | 2% | 3% | 14% | 3% | 1% | 2% | 1% |
| Other Non-Durable Medical Products | 2% | 3% | 3% | 3% | 3% | 4% | 1% | 2% | 2% |
| Government Administration | 2% | 1% | 1% | 1% | 1% | 1% | 4% | 3% | 3% |
| Net Cost of Health Insurance | 1% | 1% | 1% | 1% | 0% | 2% | 1% | 1% | 1% |
| Government Public Health Activities | 6% | 4% | 4% | 4% | 2% | 4% | 9% | 7% | 7% |
| Research | 2% | 2% | 3% | 2% | 1% | 3% | 2% | 2% | 2% |
| Structures and Equipment | 7% | 7% | 12% | 7% | 4% | 10% | 3% | 6% | 6% |

*Abbreviations*: GW = global warming; AP = acidification potential; PM = particulate matter; EP = eutrophication potential; ODP = ozone depletion potential; POP = photochemical oxidation potential (smog formation); ETP = ecotoxicity potential; HH canc. = human health cancer effects; HH non-canc. = human health non-cancer effects
