# Supplementary material for: Environmental Impacts of the U.S. Health Care System and Effects on Public Health
Source: PLoS One. 2016 Jun 9;11(6):e0157014. doi: 10.1371/journal.pone.0157014 (PMC4900601; doi:10.1371/journal.pone.0157014)
Supplement: S4 Table — (DOCX) [file pone.0157014.s005.docx]

**S4 Table. Total impacts for GHG and non-GHG categories for 2003-2013**

| **Impact Category \ Year** | **2003** | **2004** | **2005** | **2006** | **2007** | **2008** | **2009** | **2010** | **2011** | **2012** | **2013** |
| --- | --- | --- | --- | --- | --- | --- | --- | --- | --- | --- | --- |
| ***Absolute results*** |  |  |  |  |  |  |  |  |  |  |  |
| GW (kg CO_2_-e) | 5.1E11 | 5.3E11 | 5.5E11 | 5.6E11 | 5.8E11 | 6.0E11 | 6.1E11 | 6.1E11 | 6.3E11 | 6.4E11 | 6.6E11 |
| AP (kg SO_2_-e) | 2.4E9 | 2.5E9 | 2.6E9 | 2.7E9 | 2.8E9 | 2.8E9 | 2.9E9 | 2.9E9 | 3.0E9 | 3.0E9 | 3.1E9 |
| PM (kg PM_10_-e) | 8.0E8 | 8.3E8 | 8.6E8 | 8.8E8 | 9.2E8 | 9.5E8 | 9.6E8 | 9.7E8 | 9.9E8 | 1.0E9 | 1.0E9 |
| EP (kg N-e) | 7.3E7 | 7.6E7 | 7.8E7 | 8.1E7 | 8.4E7 | 8.6E7 | 8.7E7 | 8.8E7 | 9.0E7 | 9.2E7 | 9.4E7 |
| ODP (kg CFC-11-e) | 5.7E5 | 6.0E5 | 6.2E5 | 6.5E5 | 6.8E5 | 6.9E5 | 7.0E5 | 7.0E5 | 7.1E5 | 7.2E5 | 7.3E5 |
| POP (kg O_3_-e) | 3.1E10 | 3.2E10 | 3.3E10 | 3.4E10 | 3.5E10 | 3.6E10 | 3.7E10 | 3.7E10 | 3.8E10 | 3.9E10 | 4.0E10 |
| ETP (kg 2,4-D-e) | 5.4E7 | 5.6E7 | 5.7E7 | 5.9E7 | 6.1E7 | 6.2E7 | 6.4E7 | 6.4E7 | 6.5E7 | 6.7E7 | 6.8E7 |
| HH canc. (kg benzene-e) | 6.7E7 | 7.0E7 | 7.2E7 | 7.4E7 | 7.7E7 | 7.9E7 | 8.0E7 | 8.1E7 | 8.2E7 | 8.5E7 | 8.6E7 |
| HH non-canc. (kg toluene-e) | 4.1E10 | 4.3E10 | 4.4E10 | 4.6E10 | 4.7E10 | 4.8E10 | 4.9E10 | 5.0E10 | 5.1E10 | 5.2E10 | 5.3E10 |
| ***Relative results***  ***(2003 = 100)*** |  |  |  |  |  |  |  |  |  |  |  |
| GW | 100.0 | 103.5 | 107.0 | 110.2 | 114.3 | 117.4 | 118.9 | 120.3 | 122.5 | 125.8 | 128.2 |
| AP | 100.0 | 103.5 | 107.1 | 110.3 | 114.2 | 117.2 | 118.8 | 120.2 | 122.4 | 125.6 | 128.0 |
| PM | 100.0 | 103.7 | 107.4 | 110.6 | 115.2 | 118.7 | 119.6 | 121.1 | 123.7 | 127.3 | 129.7 |
| EP | 100.0 | 103.5 | 107.1 | 110.2 | 114.2 | 117.3 | 118.8 | 120.3 | 122.6 | 125.8 | 128.2 |
| ODP | 100.0 | 104.7 | 109.0 | 113.5 | 118.5 | 120.8 | 122.0 | 121.8 | 123.7 | 125.2 | 127.8 |
| POP | 100.0 | 103.8 | 107.4 | 110.7 | 115.1 | 118.5 | 119.6 | 120.9 | 123.4 | 126.8 | 129.2 |
| ETP | 100.0 | 103.1 | 106.5 | 109.4 | 112.8 | 115.6 | 117.8 | 119.4 | 121.2 | 124.3 | 126.9 |
| HH canc. | 100.0 | 103.5 | 107.0 | 110.2 | 114.0 | 117.0 | 118.9 | 120.2 | 122.3 | 125.5 | 128.1 |
| HH non-canc. | 100.0 | 103.4 | 107.0 | 110.1 | 114.0 | 117.1 | 118.8 | 120.3 | 122.4 | 125.8 | 128.4 |

*Abbreviations*: GW = global warming; AP = acidification potential; PM = particulate matter; EP = eutrophication potential; ODP = ozone depletion potential; POP = photochemical oxidation potential (smog formation); ETP = ecotoxicity potential; HH canc. = human health cancer effects; HH non-canc. = human health non-cancer effects
