# Supplementary material for: Environmental Impacts of the U.S. Health Care System and Effects on Public Health
Source: PLoS One. 2016 Jun 9;11(6):e0157014. doi: 10.1371/journal.pone.0157014 (PMC4900601; doi:10.1371/journal.pone.0157014)
Supplement: S5 Table — (DOCX) [file pone.0157014.s006.docx]

**S14 Table. Relative effect intensities of health care-related EIOLCA sectors**

|  | **Impact Category** | | | | | | | | |
| --- | --- | --- | --- | --- | --- | --- | --- | --- | --- |
| **EIOLCA sector** | GW | AP | PM | EP | ODP | POP | ETP | HH canc | HH non-canc |
| Hospitals | 62% | 71% | 37% | 62% | 6% | 39% | 41% | 53% | 50% |
| Offices of physicians, dentists, and other health practitioners | 27% | 30% | 16% | 23% | 5% | 22% | 23% | 31% | 29% |
| Healthcare and social assistance | 42% | 43% | 22% | 33% | 7% | 30% | 31% | 47% | 42% |
| Home health care services | 40% | 35% | 20% | 28% | 5% | 25% | 40% | 46% | 44% |
| Nursing and residential care facilities | 62% | 82% | 39% | 77% | 5% | 36% | 40% | 43% | 42% |
| Pharmaceutical preparation manufacturing | 59% | 69% | 41% | 56% | 39% | 51% | 19% | 49% | 38% |
| Surgical and medical instrument manufacturing | 57% | 61% | 32% | 45% | 100% | 44% | 23% | 54% | 45% |
| Surgical appliance and supplies manufacturing | 67% | 76% | 42% | 57% | 18% | 54% | 33% | 76% | 68% |
| General state and local government services | 92% | 66% | 40% | 59% | 5% | 46% | 100% | 100% | 100% |
| Insurance carriers | 11% | 13% | 7% | 10% | 1% | 10% | 8% | 10% | 10% |
| Scientific research and development services | 59% | 78% | 47% | 69% | 4% | 59% | 35% | 45% | 45% |
| Nonresidential commercial and health care structures | 100% | 100% | 100% | 100% | 8% | 100% | 29% | 68% | 74% |

Normalized by the largest value for each impact category

*Abbreviations*: GW = global warming; AP = acidification potential; PM = particulate matter; EP = eutrophication potential; ODP = ozone depletion potential; POP = photochemical oxidation potential (smog formation); ETP = ecotoxicity potential; HH canc. = human health cancer effects; HH non-canc. = human health non-cancer effects
